# Emergency Endovascular Rescue Using Fenestration for False Lumen Deployment of the Frozen Elephant Trunk in Aortic Rupture

**DOI:** 10.7759/cureus.89094

**Published:** 2025-07-30

**Authors:** Taiki Ito, Kenichiro Suno, Yasuhiro Kamikubo

**Affiliations:** 1 Cardiovascular Surgery, Kushiro City General Hospital, Kushiro, JPN

**Keywords:** aortic dissection with rupture, endovascular fenestration, false lumen deployment, frozen elephant trunk, hybrid aortic surgery

## Abstract

A 65-year-old man presented with Stanford type B aortic dissection complicated by rupture of the distal aortic arch, originating from the false lumen. Due to the short distance between the supra-aortic branches, the lack of peripheral access from malperfusion, and the invasiveness of combined arch and descending aortic replacement via left thoracotomy, emergency total arch replacement with a frozen elephant trunk was chosen to close the primary entry and control the rupture. However, intraoperative deployment of the prosthesis into the false lumen was suspected due to increasing bleeding and transesophageal echocardiographic findings. Seamless endovascular fenestration of the dissection flap was performed intraoperatively using the tail of a stiff wire under fluoroscopic guidance. By adding stent grafts to the distal true lumen, bleeding from the ruptured aorta was successfully controlled. This case highlights the feasibility and utility of a rapid, simplified intraoperative endovascular fenestration technique in a critical emergency time setting, demonstrating a life-saving strategy when the device is inadvertently deployed into the false lumen.

## Introduction

The frozen elephant trunk (FET) technique is now widely used as an effective hybrid procedure for treating complex aortic diseases involving both the arch and the descending thoracic aorta.In the past, such conditions often required two separate, highly invasive surgeries: first, replacement of the aortic arch via median sternotomy, followed by a thoracoabdominal incision to repair the descending aorta. This staged approach carried significant risks, including high complication and interstage mortality rates.The conventional elephant trunk procedure, introduced by Borst in 1983, simplified the second stage by eliminating the need for cross-clamping the descending aorta [1]. A major advancement came in 1996, when Kato and colleagues introduced the concept of the FET [2]. This technique enabled surgeons to replace the aortic arch and insert a stent graft into the descending aorta in a single operation. The stent graft provides a stable distal landing zone for future endovascular interventions.The FET technique has shown particular benefits in acute aortic dissection by re-expanding the true lumen, sealing entry tears, and promoting false lumen thrombosis. It is also effective in treating chronic aortic aneurysms and even aortic rupture involving the arch.

Despite its advantages, the technique carries certain risks. One of the most feared complications is the inadvertent deployment of the FET into the false lumen, leading to inadequate organ perfusion, continued pressurization of the false lumen, and potentially new aortic rupture.

Although multiple techniques have been described for managing such misplacements, most require complex reinterventions, often under advanced imaging guidance and in non-emergent staged settings [3,4]. By contrast, the present case involved not merely a dissection but an active rupture, which necessitated urgent decision-making and a swift, streamlined surgical response. In such life-threatening scenarios, there is no luxury of time for elaborate endovascular maneuvers or prolonged imaging evaluations. Hence, the rescue strategy must be pragmatic, quick, and executable with minimal equipment.

In this report, we present a rare and critical case of a ruptured dissecting aneurysm of the aortic arch, during which FET was unintentionally deployed into the false lumen. By performing intraoperative endovascular fenestration using only a stiff guidewire and fluoroscopy, we successfully re-established true lumen flow and sealed the rupture using additional thoracic endovascular aortic repair (TEVAR) devices. The case underscores the importance of adopting adaptable, rapid, and reproducible techniques suitable for extreme emergency situations.

## Case presentation

**Figure 1:**
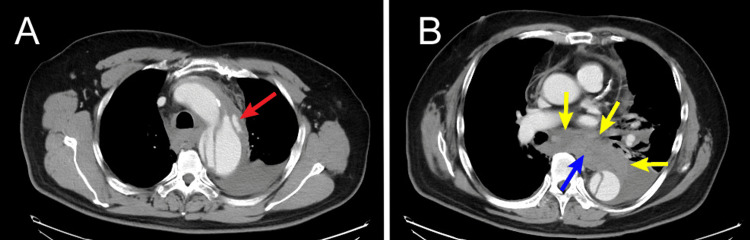
Preoperative contrast-enhanced computed tomography (A) Preoperative CECT showing a ruptured dissecting aneurysm of the distal aortic arch. The rupture site originates from the false lumen along the greater curvature, with contrast extravasation clearly visualized (red arrow). (B) Preoperative CECT demonstrating a hematoma(yellow arrow) surrounding the esophagus(blue arrow), consistent with mediastinal extension of the rupture.

A 65-year-old man with known hypertension presented to a referring hospital with acute-onset severe back pain and progressive numbness in the left lower extremity. Initial contrast-enhanced computed tomography (CECT) revealed an acute Stanford type B aortic dissection with evidence of rupture of a dissecting aneurysm in the distal aortic arch. The rupture appeared to originate from the false lumen along the greater curvature (Figure 1). Given the critical nature of the condition, he was urgently transferred to our center.

On arrival, the patient was alert but in significant distress due to persistent back pain. Blood pressure was 120/71 mmHg under continuous intravenous nicardipine hydrochloride infusion at 10 mg/h, and the heart rate was 96 beats per minute in sinus rhythm. Further evaluation confirmed an aortic rupture accompanied by a rapidly expanding mediastinal hematoma. The short interval between the supra-aortic branches precluded partial arch replacement, and the descending aorta was dilated, making it likely that the distal anastomosis would need to be performed at a lower thoracic level. In the setting of an active rupture, extensive aortic replacement was considered too invasive. It was therefore planned to control the rupture by reducing false lumen pressure through closure of the primary entry tear with a stent graft. However, due to the dissection, the true lumen of the abdominal aorta was completely occluded, and the left femoral artery, supplied by the true lumen, was pulseless due to malperfusion. There was no viable access route for TEVAR from the periphery. Therefore, it was decided to perform arch replacement with FET insertion from the central side to close the primary entry and treat the rupture.

Cardiopulmonary bypass was established with right atrial drainage and ascending aortic perfusion. Moderate hypothermia was induced, reaching a bladder temperature of 25°C. Selective antegrade cerebral perfusion was utilized along with antegrade cardioplegia for myocardial protection. A guidewire introduced from the right femoral artery was advanced to the aortic arch, but transesophageal echocardiography (TEE) revealed that it had entered the false lumen. Due to this, wire-guided FET placement was abandoned, and direct visualization was used instead. Although the misplaced guidewire might have been useful as a landmark to help distinguish between the true and false lumens, it was removed prior to circulatory arrest. When we attempted to reintroduce it during circulatory arrest, the aorta had collapsed, and the wire could no longer be advanced.

**Figure 2:**
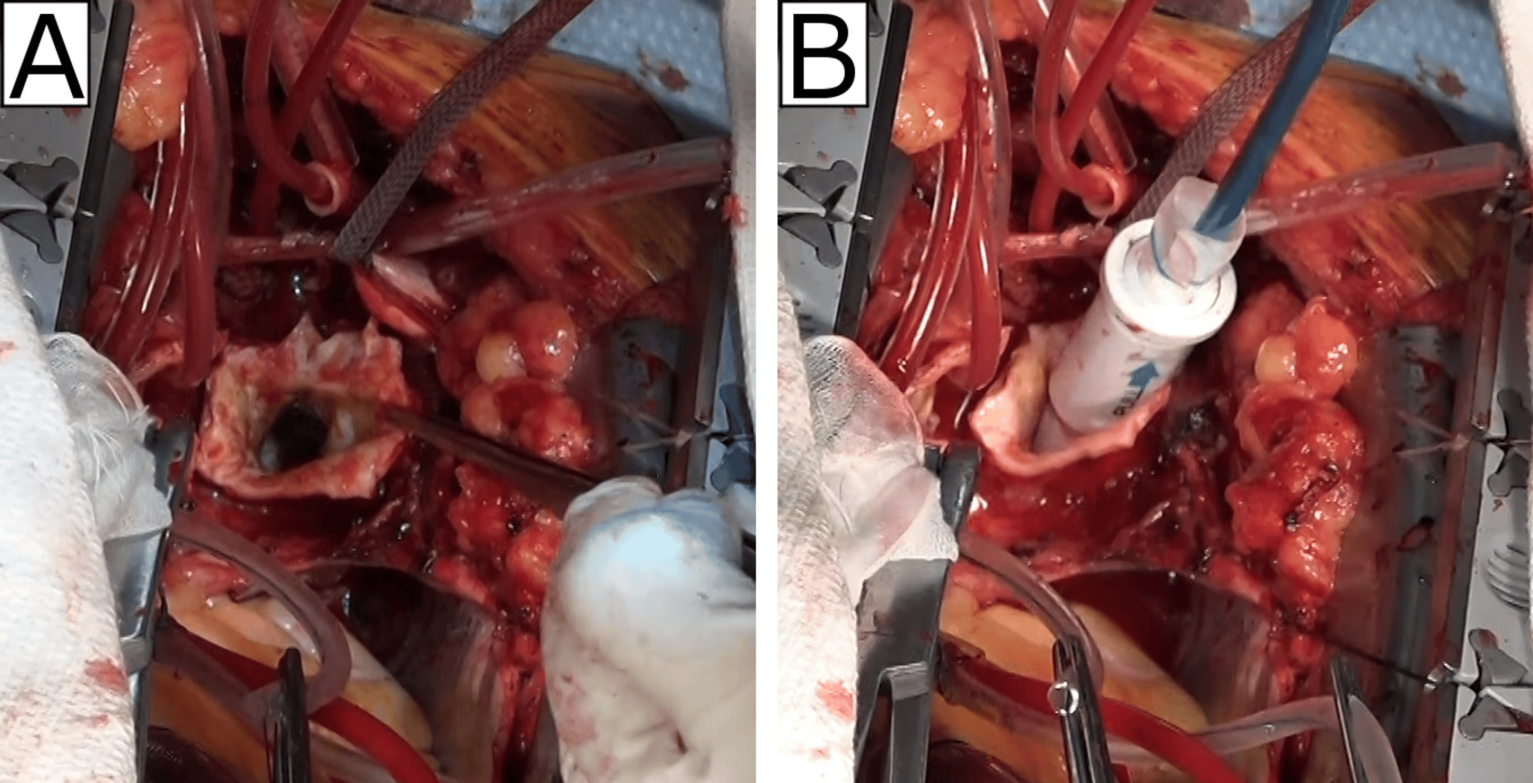
Intraoperative findings before deployment of the frozen elephant trunk (FET) (A) Surgical view after transection of the aorta at Zone 2. The intimal tear could not be identified from the operative field. (B) Introduction of the Frozenix graft into the aortic lumen. No resistance was encountered during advancement of the graft.

After transecting the aorta at zone 2 and closing the left subclavian artery, a 31 mm × 120 mm Frozenix graft (Japan Lifeline Inc., Tokyo, Japan) was inserted (Figure 2). The primary entry tear was located at the level of the tracheal bifurcation, and the dissection extended nearly circumferentially, making it difficult to discern the true lumen. Despite repeated TEE assessments and careful FET deployment, upon resuming circulation, increased bleeding from the distal end of the FET fixation site suggested misplacement into the false lumen. The left femoral pulse remained absent, and further TEE confirmed FET placement in the false lumen.

The central and supra-aortic anastomoses were completed, and the patient was gradually weaned from cardiopulmonary bypass while maintaining blood pressure at a low range to avoid further rupture.

**Figure 3:**
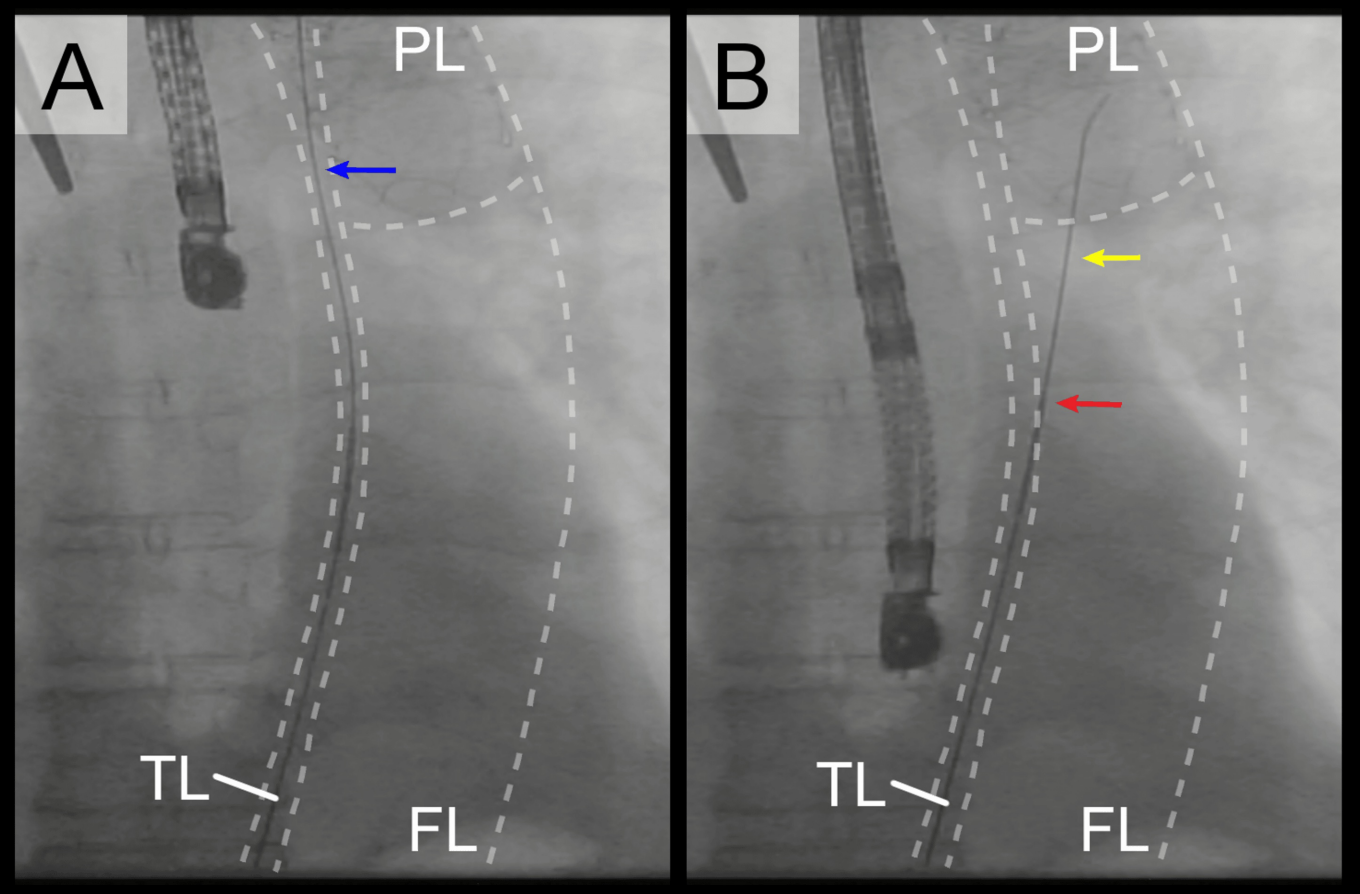
Intraoperative fluoroscopy image of the fenestration procedure (A) Fluoroscopic image showing a guidewire (blue arrow) advanced from the left femoral artery, running along the outside of the frozen elephant trunk, confirming its position within the true lumen. (B) Advancement of a curved stiff wire (yellow arrow) into the prosthetic lumen via the Kumpe Access catheter (red arrow), indicating successful endovascular fenestration. True lumen (TL), false lumen (FL), and prosthetic lumen (PL) are labeled in both panels.

From the left femoral artery, a guidewire was first advanced and observed to track along the outside of the FET, confirming that the guidewire was placed in the true lumen (Figure 3A). A Kumpe Access catheter (Cook Medical, Bloomington, IN, USA) was then positioned and left in place at the T7 level. Through this catheter, an Amplatz Ultra Stiff guidewire (Cook Medical, Bloomington, IN, USA), gently curved at its distal end, was introduced retrogradely. After slight resistance, fluoroscopy revealed that the wire was following a different path than the initial guidewire, and further advancement confirmed its entry into the FET lumen. This confirmed successful fenestration of the intimal flap and access into the false lumen (Figure 3B).

**Figure 4:**
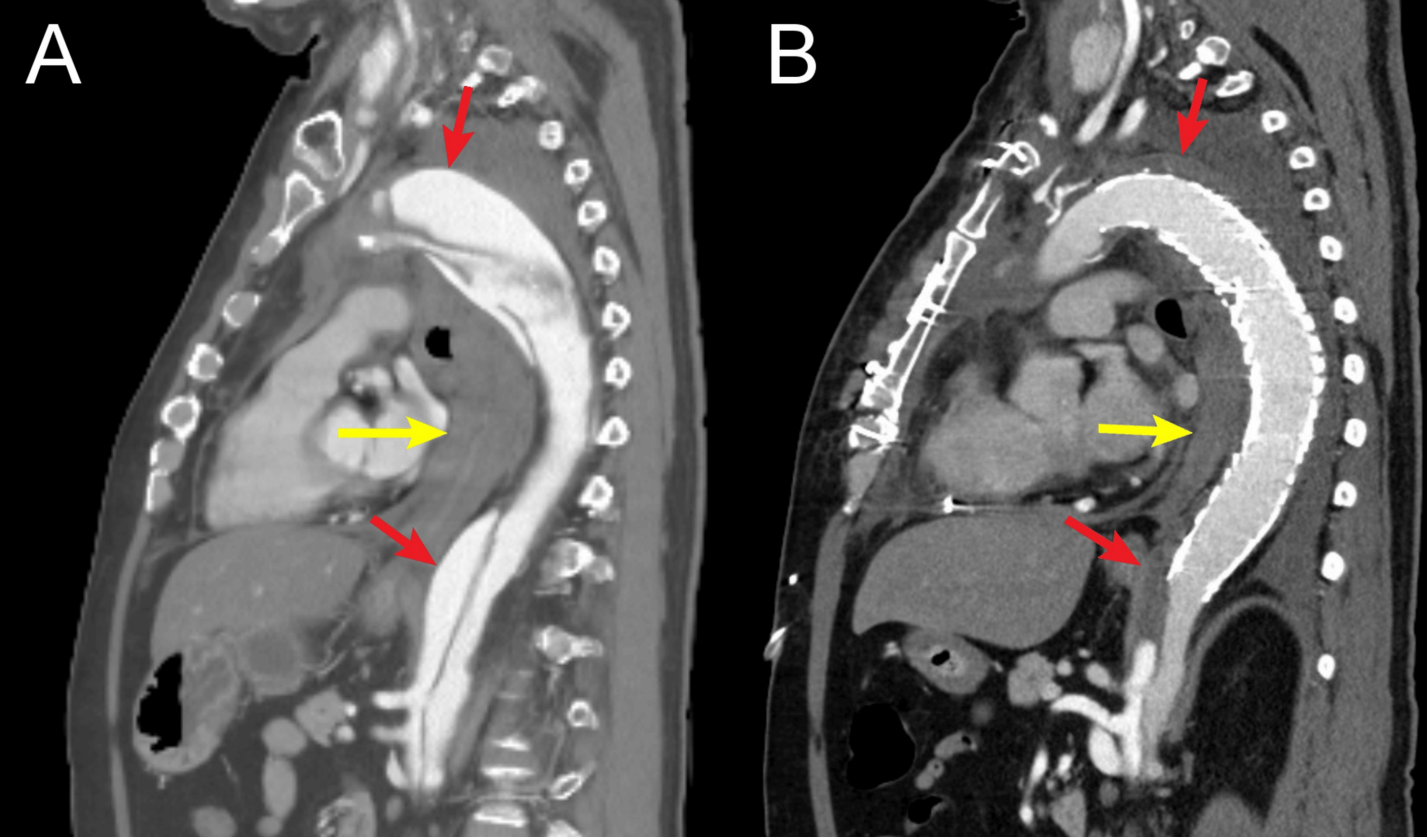
Preoperative and postoperative aortic contrast-enhanced computed tomography (A) Preoperative CECT showing a ruptured dissecting aneurysm of the distal aortic arch. The false lumen (indicated by red arrow) and a periaortic hematoma (indicated by yellow arrow) are clearly visualized. (B) Postoperative CECT demonstrating complete thrombosis of the false lumen along the entire length of the frozen elephant trunk (FET) deployment (red arrow) and a marked reduction in the periaortic hematoma (yellow arrow).

The tug-of-wire technique was employed by retrieving the wire through one of the side branches of the four-branched arch graft. This maneuver establishes a pull-through wire route from the femoral artery to the arch graft, enabling controlled traction from both ends. Following this, two conformable GORE TAG thoracic endoprosthesis (cTAG) devices (34 mm × 150 mm and 34 mm × 200 mm; W.L. Gore and Associates, Newark, DE, USA) were deployed in an overlapping fashion from the FET. The distal landing zone of the stent graft reached the T11 level, maintaining a distance of approximately 4 cm from the origin of the celiac artery, which arose at the level of L1. A significant increase in left femoral pressure was observed, and TEE reconfirmed true lumen expansion. Bleeding at the distal anastomosis visibly decreased. The patient was gradually rewarmed and successfully weaned from bypass. Postoperative recovery was smooth: the patient was extubated on postoperative day two with no neurological deficits. Follow-up CT confirmed collapse of the false lumen and shrinkage of the hematoma around the rupture site, with proper placement of the distal stent grafts in the true lumen (Figure 4).

## Discussion

The FET technique has revolutionized the management of complex aortic pathology by combining the strengths of open and endovascular approaches. However, the risk of misdeployment into the false lumen persists, especially in cases where the primary entry is not visible in the operative field and guidewire access from the periphery to the true lumen is not possible.

Various reentry techniques have been proposed to manage such complications. However, in acute rupture scenarios, prolonged procedural time and reliance on specialized tools can be detrimental.

In our patient, the FET had expanded the false lumen, leading to increased bleeding from the rupture site through the false lumen tear. In such settings, a time-efficient technique is imperative. Fortunately, during the superacute phase of dissection, the intimal flap remains thin, fragile, and compliant. As a result, even the posterior tip of a stiff guidewire has the potential to penetrate the flap without the need for sharp instruments [5].

Furthermore, since this was a case of active rupture already managed under cardiopulmonary bypass, we were able to tolerate a certain level of procedural risk. Even if a new aortic injury had occurred during fenestration, bleeding could have been controlled by blood pressure management and recovery of shed blood through the bypass circuit, as long as the cardiopulmonary bypass system remained readily available for reactivation.

Even in cases without rupture, delayed intervention for false lumen deployment carries risks of malperfusion or subsequent rupture [4]. Therefore, when false lumen placement of the FET is recognized during arch replacement, catheter-based fenestration should be proactively considered to promptly restore true lumen flow [5]. However, extension of stent grafts distally must be approached with caution, as it carries a risk of spinal cord ischemia.

In our experience, minimal equipment, a Kumpe Access catheter and a curved stiff guidewire, proved sufficient. The fluoroscopic push-through technique enabled successful fenestration, and the tug-of-wire maneuver ensured safe and accurate endograft delivery.

While this simple method was effective in our case, it may not succeed in every situation. For instance, if the intimal flap is highly mobile or not fixed, the guidewire may fail to provide adequate penetration. In such cases, tools with greater penetrative force, such as Brockenbrough needles or radiofrequency (RF) puncture systems, may be required [6,7]. Raupach et al. have reported successful fenestration using a re-entry catheter [3]. Although designed primarily for peripheral arteries, such devices may be suitable alternatives, especially when the true lumen is significantly compressed.

In addition, in cases of acute dissection where the dissection flap’s pulsatility interferes with stability, Maruyama et al. proposed temporarily stabilizing the flap by placing stent grafts proximally and distally, creating traction along the flap between the two stent grafts [4]. While this technique may increase false lumen pressure and thus must be used cautiously in rupture scenarios, it can aid in achieving precise fenestration.

As demonstrated, when stiff-wire fenestration fails in rupture cases, it is essential to promptly transition to alternative strategies based on the available tools and the operating surgeon's expertise.

Although we did not employ intravascular ultrasound (IVUS), it is important to note that IVUS is extremely useful for confirming true lumen wire placement throughout its entire course. After circulatory arrest, imaging quality may deteriorate due to a collapsed aorta; however, IVUS may still help prevent inadvertent deployment of the FET into the false lumen. Moreover, IVUS can also be beneficial after false lumen deployment has occurred, particularly when used in conjunction with other devices to guide controlled fenestration of the intimal flap. In the present case, TEE was limited due to a hematoma surrounding the esophagus. In such rupture cases, IVUS may provide especially valuable information.

This case illustrates that, even under the extreme stress of active rupture and limited imaging capacity, a simplified, minimally invasive endovascular salvage technique is both practical and effective. Its simplicity, reproducibility, and adaptability make it a valuable option in the hybrid surgical management of complex and acute aortic disease.

## Conclusions

Intraoperative endovascular fenestration is a feasible, effective, and potentially life-saving technique when inadvertent false lumen deployment of the FET occurs during emergency aortic arch surgery. Particularly, in rupture settings, where time and visibility are limited, a simplified fluoroscopy-guided strategy using a curved stiff wire can enable rapid reentry into the true lumen. The optimal fenestration method should be selected based on the specific morphology of the dissection and the time constraints present in each case.

## References

[1] Borst HG, Walterbusch G, Schaps D (1983). Extensive aortic replacement using "elephant trunk" prosthesis. Thorac Cardiovasc Surg.

[2] Kato M, Ohnishi K, Kaneko M, Ueda T, Kishi D, Mizushima T, Matsuda H (1996). New graft-implanting method for thoracic aortic aneurysm or dissection with a stented graft. Circulation.

[3] Raupach J, Chovanec V, Kozakova V, Vojacek J (2020). Endovascular fenestration of aortic dissection membrane after failed frozen elephant trunk procedure. Eur J Cardiothorac Surg.

[4] Maruyama T, Enomoto Y, Ito C (2020). Endovascular aortic fenestration and Stent graft placement after inadvertent false lumen deployment of a frozen elephant trunk. J Jpn Coll Angiol.

[5] Witcher AC, Meers B, Lewis CT, Beck AW, Eudailey KW (2023). Rescue of false lumen frozen elephant trunk deployment intraoperatively. Ann Thorac Surg.

[6] Wong RH, Yu PS, Kwok MW, Chow SC, Ho JY, Underwood MJ, Yu SC (2017). Endovascular fenestration for distal aortic sealing after frozen elephant trunk with thoraflex. Ann Thorac Surg.

[7] Plotkin A, Hanks SE, Han SM, Fleischman F, Weaver FA, Magee GA (2019). Endovascular septal fenestration using a radiofrequency wire to salvage inadvertent false lumen deployment of a frozen elephant trunk stent graft. J Vasc Surg Cases Innov Tech.

